# Supplementary material for: Characterization of A-to-I Editing in Pigs under a Long-Term High-Energy Diet
Source: Int J Mol Sci. 2023 Apr 27;24(9):7921. doi: 10.3390/ijms24097921 (PMC10178050; doi:10.3390/ijms24097921)
Supplement: Supplementary file 1 [file ijms-24-07921-s001.zip › ijms-2290166-supplementary captions.pdf]

Supplementary Tables:

Table S1. Sample information.

Table S2. A-to-I editing levels across all samples.

Table S3. Gene annotation of A-to-I editing sites.

Table S4. Gene annotation of specific and different editing A-to-I sites.

Table S5. Functional enrichment of specific and different editing A-to-I sites.

Table S6. Functional A-to-I editing sites in gene features.

Table S7. Protein coding change of A-to-I editing sites.

Supplementary Figures:

Figure S1. A-to-I editing across pig genome. **a.** Frequency distribution of each type of RNA editing site across pig genome.**b.** Editing level distribution of each type of RNA editing site across pig genome. **c.** Frequency distribution of A-to-I editing sites across the chromosomes. **d.** Frequency distribution of A-to-I editing sites in the chromosomes. **e.** Count distribution of the distance of sites to sites. **f.** Frequency distribution of A-to-I editing levels. **g.** Frequency distribution of A-to-I editing detected samples.

Figure S2. Count of A-to-I editing across genes and pig tissues. **a.** Count of A-to-I editing sites in each gene biotype. **b.** Count of A-to-I editing sites in each gene feature. **c.** Upset plot of A-to-I editing sites in 3 tissues. **d.** Upset plot of A-to-I editing sites in 6 gut segments.

Figure S3. Count statistics and gene enrichment of functional A-to-I editing site. **a.** Proportion of functional A-to-I sites across tissues. **b.** Venn plot and upset plot of functional A-to-I sites for 3 pig tissues and 6 gut segments. **c.** Gene enrichments of functional A-to-I sites in 3 pig tissues. **d.** Gene enrichments of functional A-to-I sites in 6 gut segments.

Figure S4. Illustration of the gene region of two shared function A-to-I editing sites. **a.** Gene location of 7:24969309 function A-to-I editing site. **b.** Gene location of 17:51259503 function A-to-I editing site.

Figure S5. Count and fold enrichment of functional A-to-I editing sites in each gene biotype and gene feature. **a.** Count of functional A-to-I editing sites in each gene biotype for each type of functional A-to-I editing site. **b.** Fold enrichment of functional A-to-I editing sites in each gene biotype for each type of functional A-to-I editing site. **c.** Count functional A-to-I editing sites in each gene feature for each type of functional A-to-I editing site. **d.** Fold enrichment of functional A-to-I editing sites in each gene feature for each type of functional A-to-I editing site.
