# Supplementary figures and images for: Characterization of A-to-I Editing in Pigs under a Long-Term High-Energy Diet
Source: Int J Mol Sci. 2023 Apr 27;24(9):7921. doi: 10.3390/ijms24097921 (PMC10178050; doi:10.3390/ijms24097921)

**a**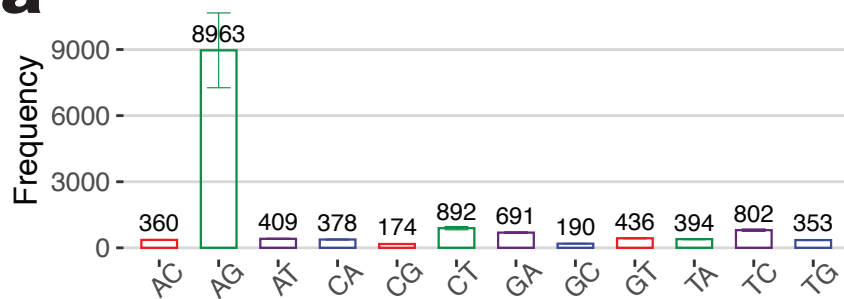**b**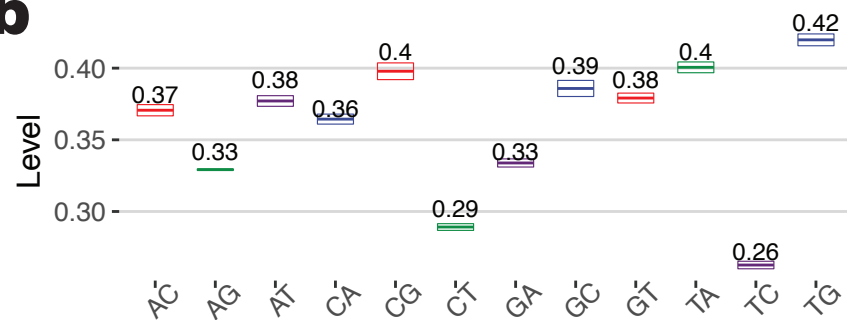**c**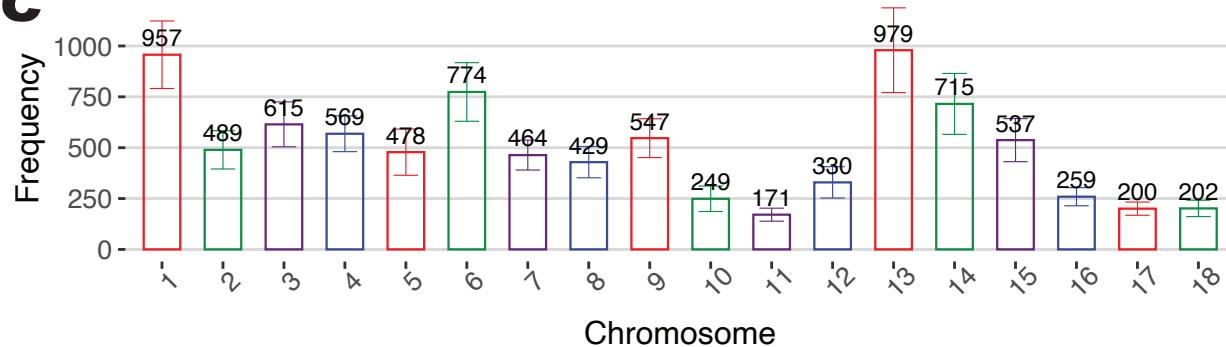**d**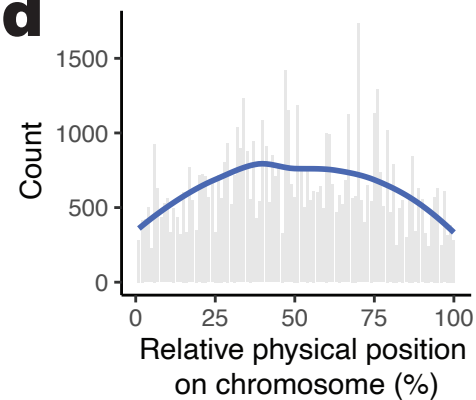**e**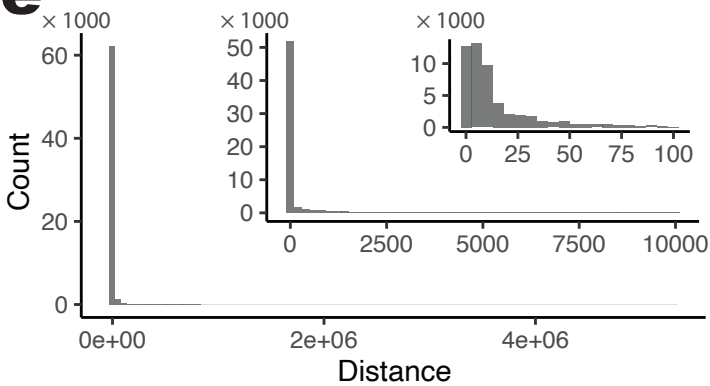**f**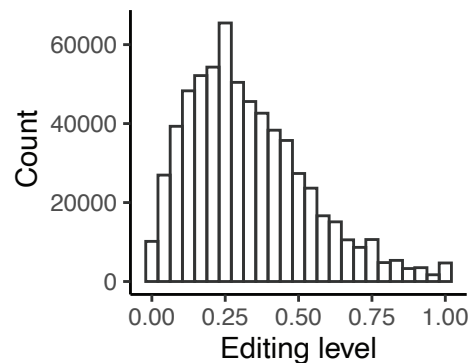**g**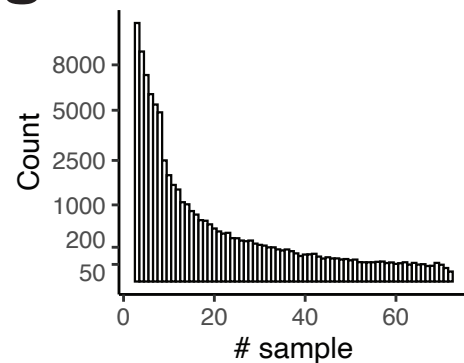

Supplement: Supplementary file 1 [file ijms-24-07921-s001.zip › Figure S1.pdf]

**a**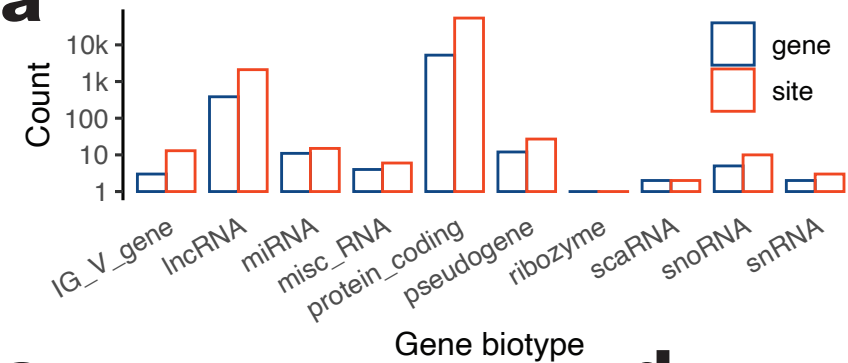**b**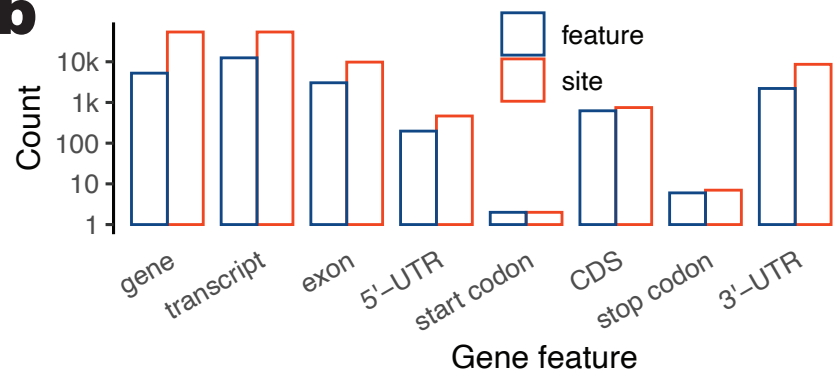**c**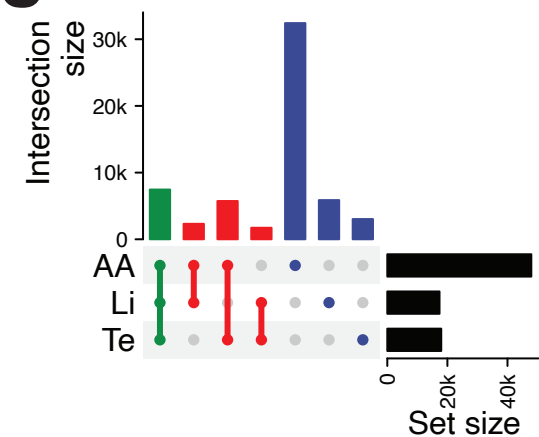**d**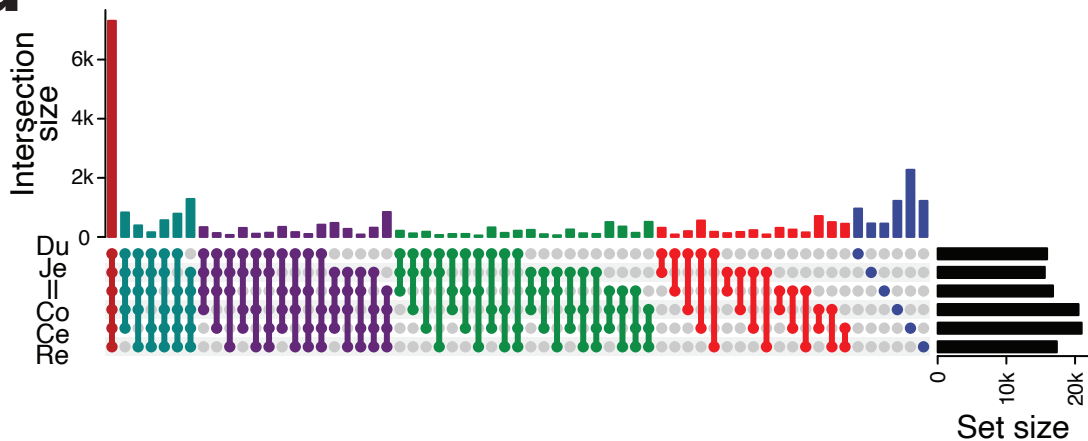

Supplement: Supplementary file 1 [file ijms-24-07921-s001.zip › Figure S2.pdf]

**a**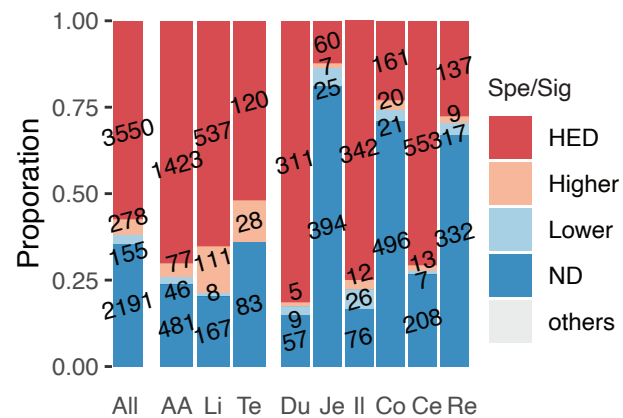**b**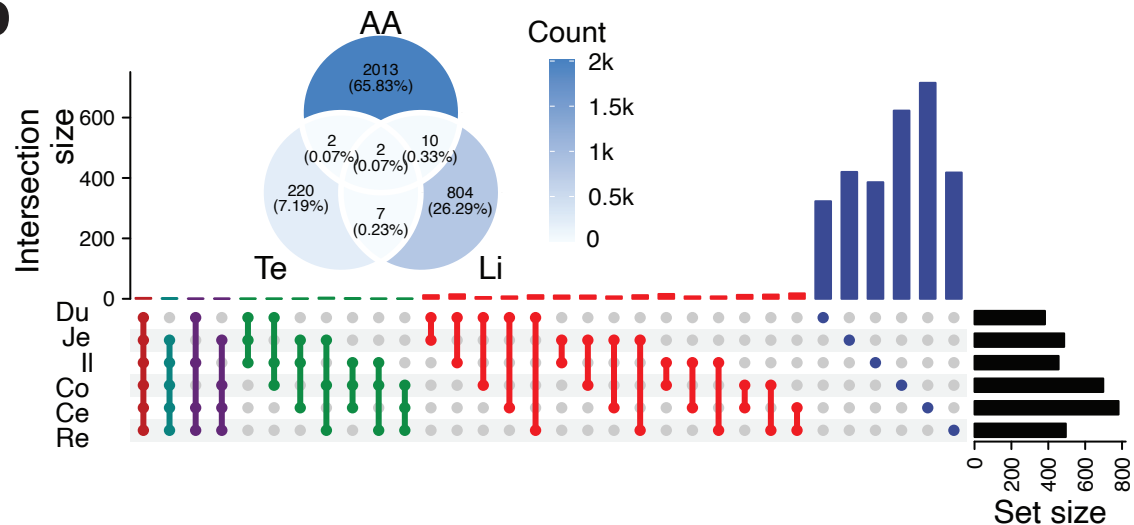**c**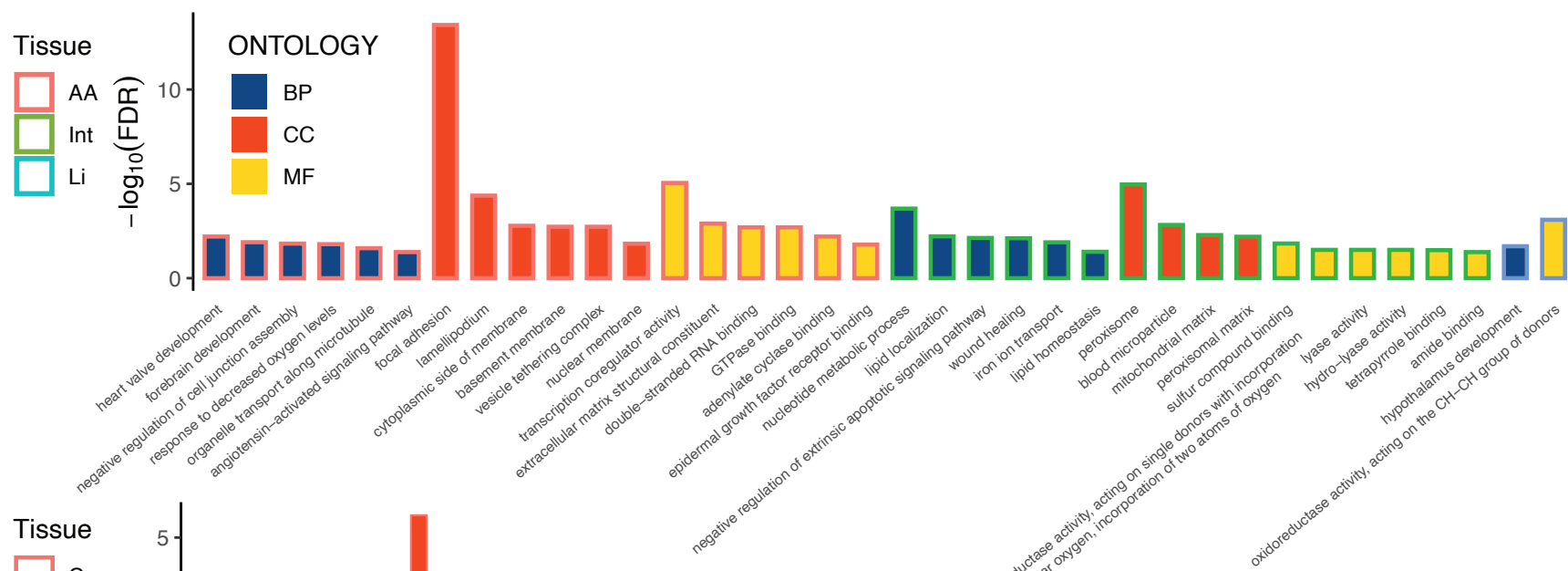**d**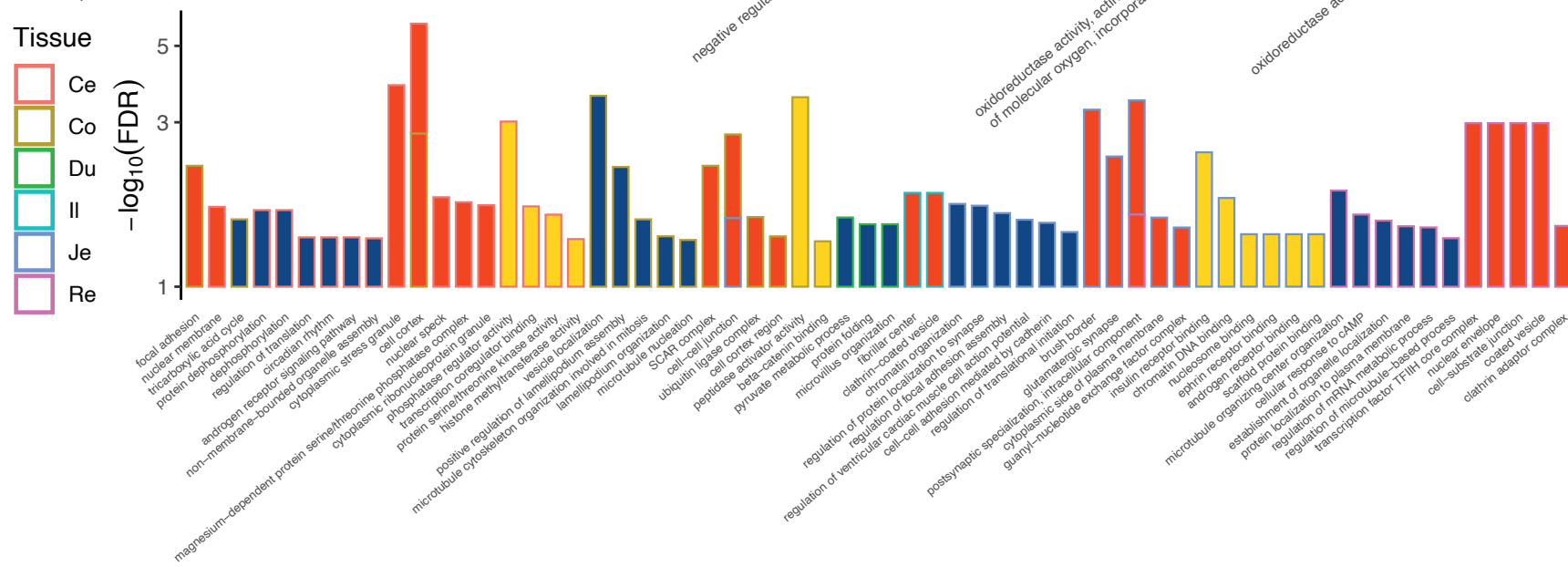

Supplement: Supplementary file 1 [file ijms-24-07921-s001.zip › Figure S3.pdf]

**a**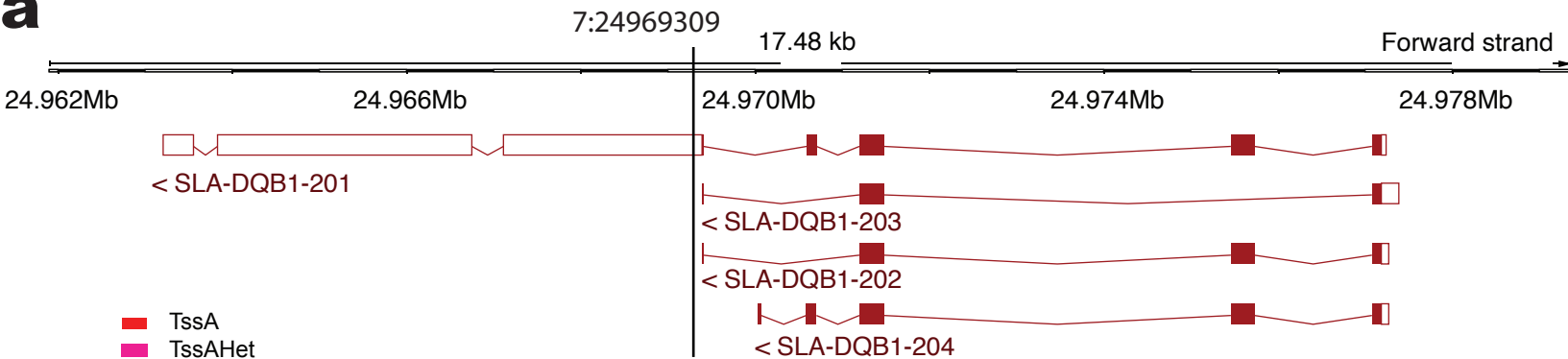**b**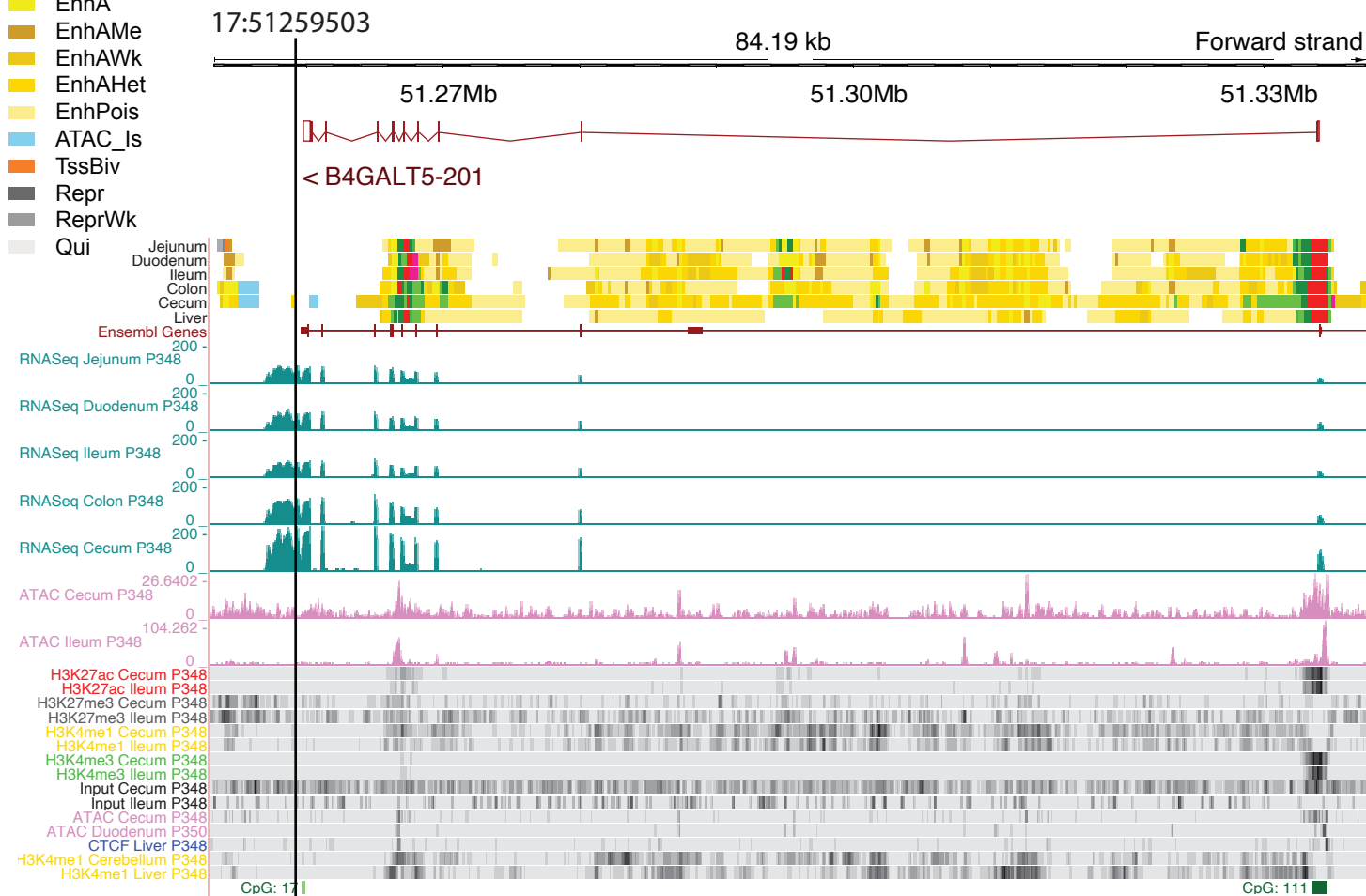

Supplement: Supplementary file 1 [file ijms-24-07921-s001.zip › Figure S4.pdf]

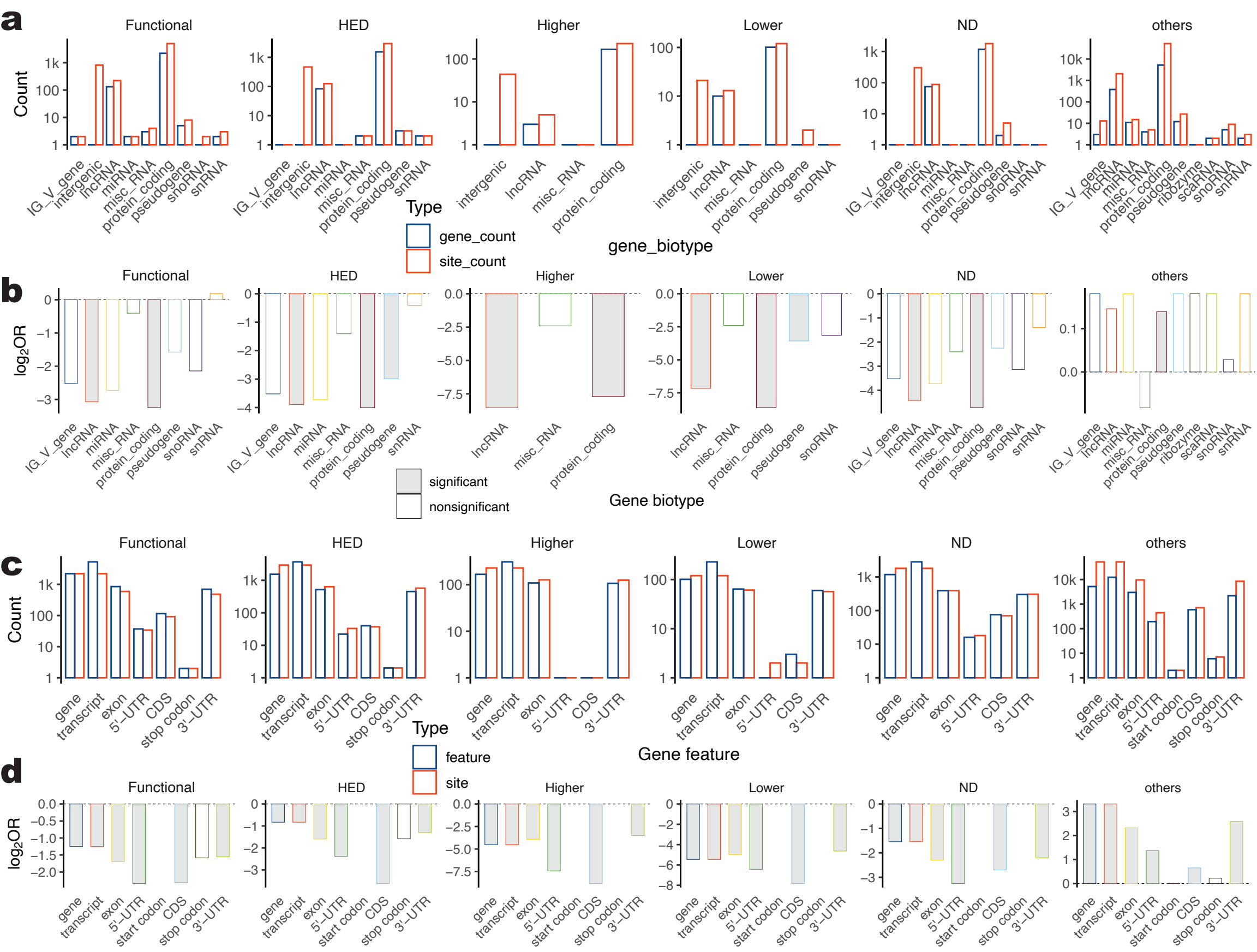

Supplement: Supplementary file 1 [file ijms-24-07921-s001.zip › Figure S5.pdf]
